# Robustness of Deep Networks for Mammography: Replication Across Public Datasets

**DOI:** 10.1007/s10278-023-00943-5

**Published:** 2024-01-10

**Authors:** Osvaldo M. Velarde, Clarissa Lin, Sarah Eskreis-Winkler, Lucas C. Parra

**Affiliations:** 1 The Department of Biomedical Engineering, The City College of New York, 10030 New York, NY USA; 2 Department of Radiology, Memorial Sloan Kettering Cancer Center, 10065 New York, NY USA

**Keywords:** Deep learning, Diagnosis, Breast cancer, Mammography

## Abstract

Deep neural networks have demonstrated promising performance in screening mammography with recent studies reporting performance at or above the level of trained radiologists on internal datasets. However, it remains unclear whether the performance of these trained models is robust and replicates across external datasets. In this study, we evaluate four state-of-the-art publicly available models using four publicly available mammography datasets (CBIS-DDSM, INbreast, CMMD, OMI-DB). Where test data was available, published results were replicated. The best-performing model, which achieved an area under the ROC curve (AUC) of 0.88 on internal data from NYU, achieved here an AUC of 0.9 on the external CMMD dataset (*N* = 826 exams). On the larger OMI-DB dataset (*N* = 11,440 exams), it achieved an AUC of 0.84 but did not match the performance of individual radiologists (at a specificity of 0.92, the sensitivity was 0.97 for the radiologist and 0.53 for the network for a 1-year follow-up). The network showed higher performance for in situ cancers, as opposed to invasive cancers. Among invasive cancers, it was relatively weaker at identifying asymmetries and was relatively stronger at identifying masses. The three other trained models that we evaluated all performed poorly on external datasets. Independent validation of trained models is an essential step to ensure safe and reliable use. Future progress in AI for mammography may depend on a concerted effort to make larger datasets publicly available that span multiple clinical sites.

**Supplementary Information:**

The online version contains supplementary material available at 10.1007/s10278-023-00943-5.

## Introduction

Women in the USA have a 13% lifetime risk of breast cancer [1]. Breast cancer mortality can be substantially reduced by early detection with mammography before signs are apparent on physical exam [2]. Annual screening mammography for early cancer detection is recommended for average-risk women beginning at age 40, and more than 40 million mammograms are performed each year in the USA alone [3].

Two decades ago, computer-aided detection (CAD) technology was introduced to aid in the clinical detection of breast cancer on mammography, with disappointing results [4, 5]. The performance of CAD tools can often achieve high sensitivity, but this often comes at the expense of a relatively high false-positive rate [4]. However, with recent advances in deep learning (DL) technology, newer models have been developed, bolstered by large datasets of labeled images from breast cancer screening programs. These newer models have been shown to perform at or even beyond the level of radiologists [6–9].

**Fig. 1:**
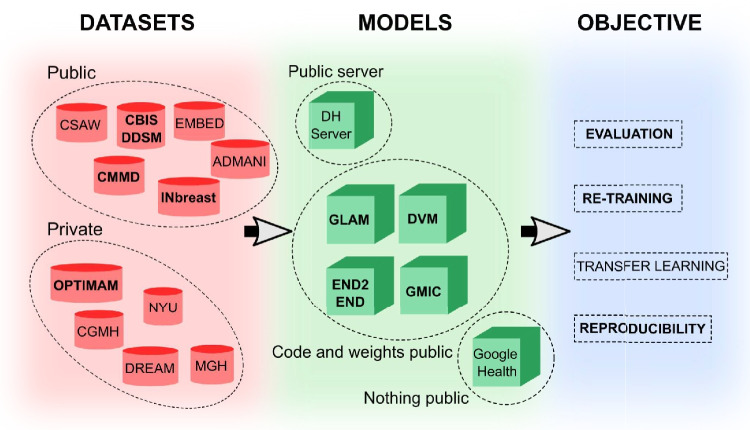
Current availability of mammography datasets and deep-learning models. In the literature, several datasets, models, and research objectives are presented [7, 9–16]. In this work, we focus on some of them (bold font). After training a model with a large dataset A, research objectives may include evaluation, re-training, transfer learning, or testing for reproducibility on external datasets. For evaluation, one requires both the model architecture and the model weights to determine how well it performs on a smaller dataset B. In cases where dataset B is large, re-training the model from scratch using just the model architecture may be possible. For transfer learning, both the architecture and trained weights of a model are needed to use it as a feature extractor, such as in segmentation networks. Reproducibility requires access to the original dataset A and the model architecture to reproduce published results

However, many of these published efforts were tested only on internal data and have not made their trained models available. This precludes independent testing of the models, which would provide an important additional layer of validation. It also impedes further development that could spur progress in the field. Many groups also keep their imaging data private, which does not benefit the broader research community. A detailed description of the model architecture, ideally specified in open-source code, along with pre-trained model parameters, should be standard in the field. Figure 1 represents the current situation in terms of the public release of datasets and models at the start of this study. There are a few releases of small test sets, but these are not large enough to train models from scratch [10–12]. Fortunately, some teams have made their model architectures and trained parameters available [7, 9, 13–16]. Teams have also released limited mammography data for research purposes [10–12].

In this work, we seek to validate available state-of-the-art DL models for mammography on publicly available datasets. To do so, we tested four trained models on four external datasets. For the best-performing model, we analyze tumors that were missed by the DL model to explore sources of error in DL-based cancer detection.

## Materials and Methods

We assessed AI models that were made available through the public release of code and trained model parameters. This includes three models from Krzysztof Geras’ group at NYU (GLAM [16], GMIC [13], DMV [15]) and one from Li Shen’s group at MSSM (End2End [7]). These models had been trained and evaluated using a combination of public and private databases. For each of the four models, we evaluated performance on each of the three publicly available datasets: INbreast [10], CBIS-DDSM [11], and CMMD [12]. Finally, we evaluated performance of the best-performing model using the OPTIMAM project OMI-DB dataset [17].

### Description of Available Datasets

**Fig. 2:**
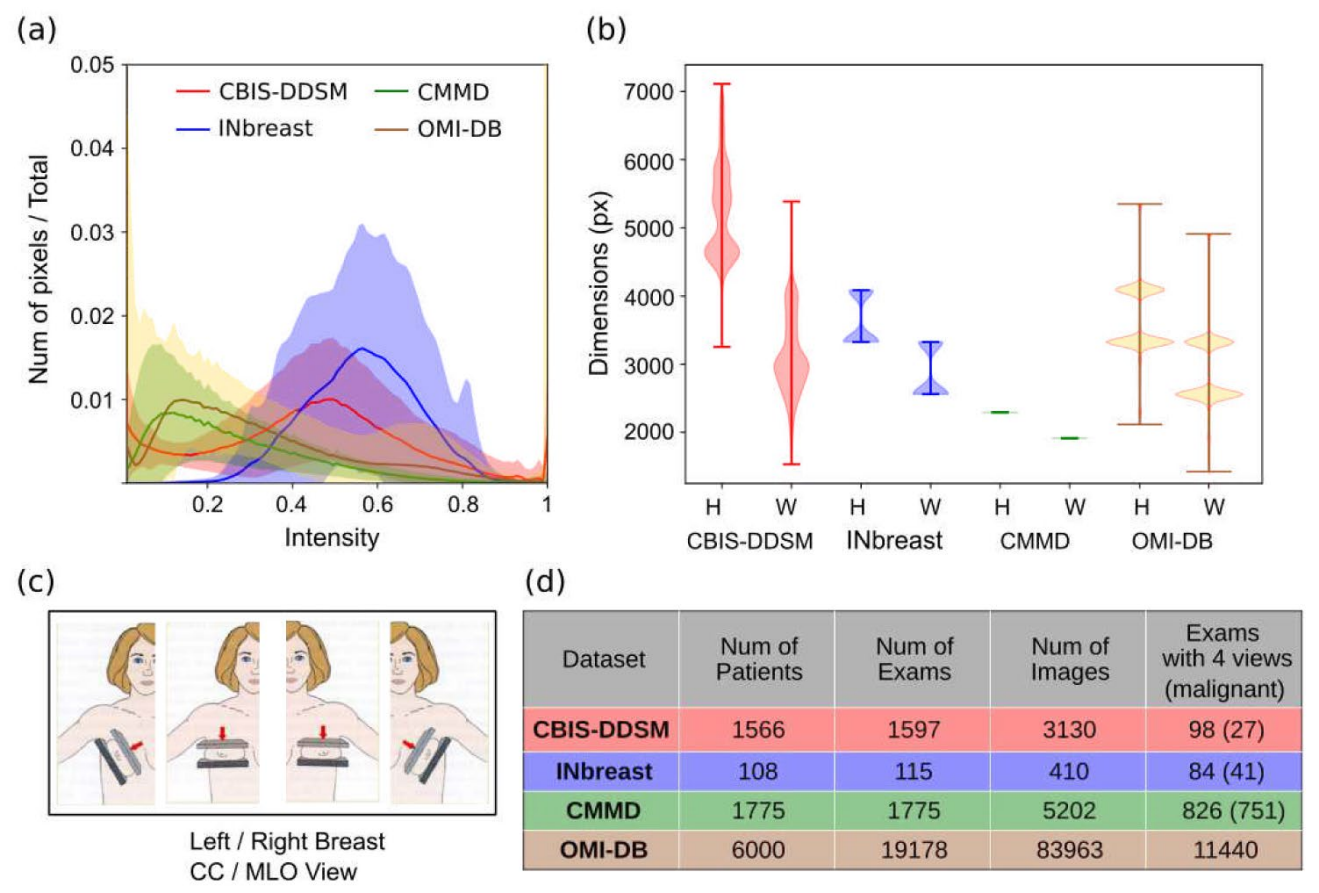
Statistics of the available datasets used in this work. **a** Histogram of pixel intensities averaged over all images (solid lines). Shaded areas cover the standard deviation of histograms over all images in a dataset. All datasets have 8-bit images that have been rescaled in intensity to the range of 0 to 1. **b** Distribution across images for image height (H) and width (W). **c** Standard mammogram views: craniocaudal (CC) and mediolateral oblique (MLO). **d** Number of patients, exams, and images available for each dataset. For the last column, the number of exams with at least one malignant lesion is indicated in parentheses. For the OMI-DB dataset, Table 2 provides detailed information on the distribution of exams

Four datasets were compiled: INbreast [10], CBIS-DDSM [11], CMMD [12], and OMI-DB [17]. Figure 2 shows some characteristics and statistics of each dataset such as the distribution of pixel intensity (Fig. 2a) and the distribution of image sizes (Fig. 2b). The distributions presented are for the images before applying the specific preprocessing steps of each model. In addition, the table (Fig. 2d) indicates the number of patients, exams, and images for each dataset. Each exam can contain up to 4 images: left and right breast in MLO and CC views (Fig. 2c). The OMI-DB dataset contains multiple screening exams per patient and the opinions of different radiologists [17]. In this dataset, each exam was evaluated by two to five randomly selected radiologists, and a consensus opinion was generated. Only the 1st radiologist (who is not always the same person) is an independent reading as readers have access to the preceding opinions. The consensus opinion guides the decision on whether to recall the patient for further assessments, such as a biopsy, which may result in a pathology finding. In total, the dataset includes 4000 patients with malignant findings across any of the screening exams. We excluded exams that did not have a subsequent 3-year screening. We also excluded incomplete exams where one of the four standard views was missing or images that were not intended for presentation to a radiologist (e.g., images labeled “for processing” intended for automatic image processing such as segmentation). In addition, we discard images that have artifacts. We were left with 5935 patients and a total of 11,440 exams with a 3-month follow-up, which are predominantly negative despite the enriched number of cases because there are several negative exams prior to a malignant finding. The Supplementary Information provides additional details about each dataset.

### Model Structure and Evaluation

We studied different models (End2End, GLAM, GMIC, and DMV) with various architectures and training processes.

There are notable differences between the general structure of the models. The End2End model only consists of a convolutional network. The DMV model consists of 4 convolutional networks working in parallel (one CNN per view) and then the outputs are combined. The GLAM and GMIC models contain three modules. The global module is a memory-efficient CNN that extracts the global context and generates prominence maps that provide an approximate location of possible benign/malignant findings. On the other hand, the local module is a CNN with a greater capacity to extract visual details of regions of interest (patches) and then condense this information using an attention or aggregation mechanism. Finally, the fusion module combines the representation vectors of the global and local modules to produce a combined prediction. The main difference between GLAM and GMIC lies in the global module. On the one hand, in GLAM, the global module provides a pyramidal hierarchy of multi-scale feature maps when processing an input image. In contrast, GMIC only provides a single-scale feature map. More details are explained in the Supplementary Information.

Each model defines a specific way to preprocess the images before feeding them into the neural network. The preprocessing steps may include the following: (1) a procedure to crop all valid mammography images, ensuring they only contain the relevant breast regions; (2) a data augmentation technique; and (3) normalization of pixel intensities. For specific information on each step of the preprocessing, we recommend reading the articles associated with each model and the report presented by Wu et al. [18].

Additionally, the models differ in the output they generate:

1. A continuous variable $y$ between 0 and 1 capturing the probability of “cancer” vs “normal.” The End2End model uses this type of output.
2. Two continuous variables capturing the probability of malignant lesions as well as benign lesions (i.e., $[{y}_{m},{y}_{b}]$) as the image may contain both, either or none. This is the approach implemented in GLAM and GMIC models.
3. Four continuous variables capturing the probability of the presence of malignant and benign lesions for each breast (i.e., $[{y}_{mL},{y}_{mR}, {y}_{bL}, {y}_{bR}]$). The DMV model uses this type of output.

In this work, we only evaluated the output variable that estimates the likelihood of a malignant lesion. The likelihood that there is a benign lesion is ignored in the evaluation. The networks either take an individual image as input producing an output regardless of what view or breast side was provided (End2End, GLAM, GMIC), or the network takes all 4 images as input producing an output for each of the two breasts (DMV), i.e., combining information from CC and MLO views. Where images are processed individually, the prediction of the network is computed for each breast as the average output for CC and MLO views (note that exams occasionally are missing one of the two views in all datasets used here).

### Confidence Intervals

**Fig. 3:**
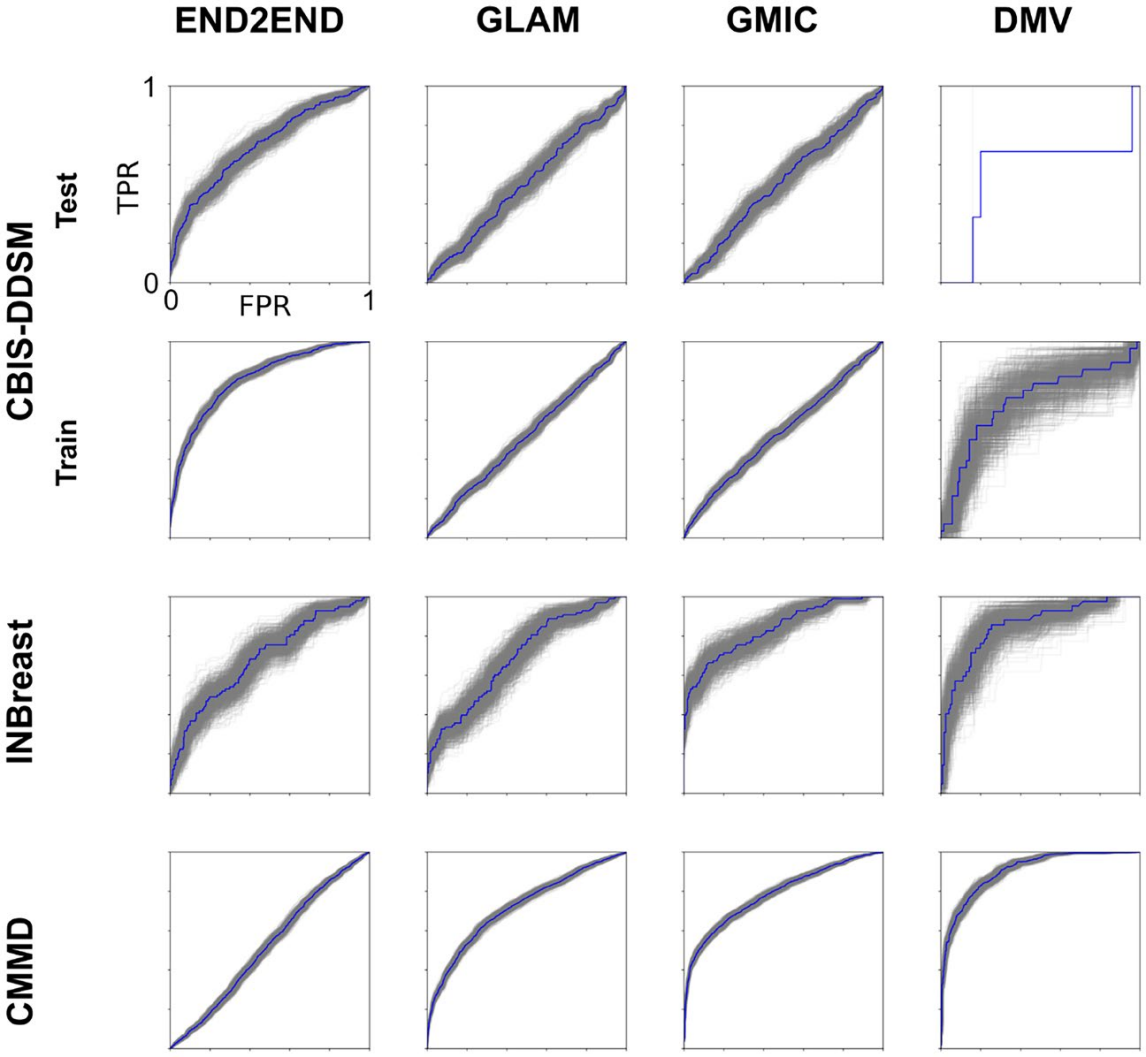
ROC curves for various published models and datasets. For each model, we calculated predictions for all breasts in the corresponding dataset. Using the set of predictions and labels, we determined the ROC curve (blue), i.e., the true-positive rate (TPR) vs. the false-positive rate (FPR). Gray curves indicate 1000 bootstrap samples. For the DMV model on the CBIS-DDSM, we did not perform bootstrapping as there were only 4 cancers with four views

All confidence intervals are estimated using a bootstrap procedure. Bootstrapping resamples predictions and labels with replacement 1000 times and calculates ROC curves for these newly sampled sets (e.g., gray curves in Fig. 3).

## Results

### Independent Validation of Four Publicly Available DL Models

The ROC curves evaluated on individual breasts are shown in Fig. 3 for all combinations of models and public datasets. The area under the curve is summarized in Table 1, which also lists performance as reported in the original publications, on private datasets. The general observation is that the previously published results are all numerically higher than the performance of the same models on the public datasets (statistical comparison is not feasible as full ROC curves from these published results are not available). There are only two exceptions to this. The End2End model was tested originally on a subset of the public CBIS-DDSM data (Table 1, bottom row), which we reproduce here exactly (not shown). However, on the full CBIS-DDSM test data, performance is numerically lower (Table 1, first blue row; we omit statistical comparisons as we are uncertain whether the test set was excluded from training). The other exception is the DMV model (right column), which seems to generalize well to the CMMD and INbreast data (second and third blue row) with performance numbers comparable to the previously published results (0.9, 0.85 here vs the published 0.88). The DMV performed better than the closest competitor (GMIC) for the CMMD data (Delong test, *z* = 6.47, *p* = 10^−10^, 826 exams) but not the INbreast data (Delong test, *z* = 0.57, *p* = 0.6, 84 exams).

**Table 1:** Area under the ROC (AUC) for various public models and datasets. Results in blue are obtained here with publicly available data and networks evaluated on individual breasts. For reference, results from previous publications are shown in the lower gray row. For DMV, there is an option of operating on individual images or generating predictions jointly using 2 views of both breasts. (*) In this work, we evaluate the models in the test set predefined by the CBIS-DDSM dataset; however, Shen et al. [7] evaluates the model in a subset defined by the authors. In Stadnick et al. [19], similar results across models and datasets are shown in this table

		Model				
Properties	Name	**End2End**	**GLAM**	**GMIC**	**DMV**	
	Group	MSSM	NYU	NYU	NYU	
	Input	1 image: CC and/or MLO of one breast				4 images
Datasets evaluated here	CBIS- DDSM	**0.70** ± 0.02	0.50 ± 0.02	0.51 ± 0.02	0.54 ± 0.02	0.56 ± 0.01
	INbreast	0.67 ± 0.03	0.70 ± 0.03	0.84 ± 0.02	0.75 ± 0.03	**0.85** ± 0.04
	CMMD	0.53 ± 0.01	0.76 ± 0.01	0.80 ± 0.01	0.79 ± 0.01	**0.90** ± 0.01
Published previously	Performance (data, year)	0.85^7^ (CBIS-DDSM*, 2019)	0.82^16^ (NYU, 2021)	0.91^13^ (NYU, 2020)	NA	0.88 ^15^ (NYU, 2020)

The values in bold font indicate the best performance for each dataset

### Performance of DMV in the OMI-DB Database

For the OMI-DB database, we measured the diagnostic accuracy of the model and the radiologist’s opinions based on pathology results, either immediately following the exam (0–3 months) or after some follow-up period (1 year, 2 years, 3 years). We also determine the ability to predict the outcome of the next 3-year screening exams. Table 2 shows a breakdown of when tumors were detected following an exam.

**Table 2:** OMI-DB data: number of breast examined and pathology outcomes at various follow-up intervals

Radiologist consensus opinion	Number of breasts examined	Number of breasts with malignant pathology (cumulative)				
		0–3 months screen detect	0–1 y interval	0–2 ys interval	0–3 ys interval	0–3 years + screen detect
Normal	17,199	43	69	123	236	1503
Benign	94	36	36	37	37	38
Uncertain	5265	3780	3782	3784	3789	3826
Suspicious	181	121	121	121	122	122
Malignant	87	85	85	85	85	85

**Fig. 4:**
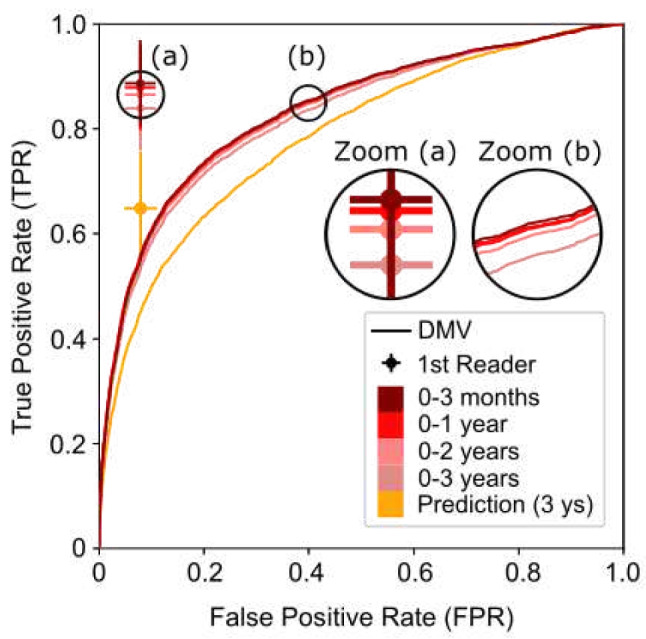
Performance of the DMV model in diagnosis and prediction for network and 1st radiologists reader

We focus the evaluation on the best-performing public model (DMV) (Fig. 4). The pre-trained DMV achieved an AUC of 0.84 ± 0.01 (95% CI) at the diagnosis task regardless of the follow-up period (Table 3). For the 3-year prediction, the AUC is 0.79 ± 0.01. This is evidently a more difficult task with a drop in performance (which we also see in the results of Table 4). Table 3 also compares the AUC curves for the DMV network with published results of networks evaluated on various releases (subsets) of the OMI-DB database.

**Table 3:** Results on OMI-DB database. Our work (blue) evaluated the publicly available DMV model using heatmaps (see Fig. 5d), while Standick et al. (gray row) evaluated the DMV model without heatmaps. Other published results (gray rows) used networks that are not publicly available for validation (gray) and have used different releases of the OMI-DB. Follow-up “3 years + ” indicates the task of predicting all interval cancer including screen-detected cancers at the 3rd year of scheduled screening. Follow-up “1–2 years*” indicates the task of prediction of screen-detected cancers detected in the interval between months 12 to 24 after the screening. **The results of Deep Health are limited to mammograms collected with a Hologic scanner

**Author**	**Group (Model)**	**Follow-up**	**AUC**	**Number of positive exams**	**Num of exams**
Here	DMV	3 months	0.84 ± 0.01	3965	11,440
		1 year	0.84 ± 0.01	3997	
		2 years	0.84 ± 0.01	4053	
		3 years	0.84 ± 0.01	4168	
		3 years +	0.79 ± 0.01	5457	
Stadnick et al. [19]	NYU-DMV	3 months	0.767	1023	11,633
Kim et al. [20]	Yonsei Univ	1 year	0.938	218	654
Pedemonte et al. [21]	Whiterabbit AI Inc	2 years	0.947	561	1877
		3 years	0.894	595	
McKinney et al. [8]	Google Health	3 years	0.889	414	25,856
Lotter et al. [9]	Deep Health**	3 months	0.963	1205	2743
		1 year	0.959	1243	
		1–2 years*	0.744	68	NA

Note that the immediate diagnosis outcomes have a confirmation bias because the consensus opinions guide biopsy decisions and thus normal/benign outcomes depend on their reading. This is also true for the 1st reader because their opinion is included in the consensus reading. However, we can evaluate the radiologist’s opinion at future time points that include cancers detected during the subsequent 3-year screening interval. Sensitivity (true positive rate) and specificity (1-false positive rate) are summarized in Table 4 for the 1st reader as well as the DMV network. We also evaluate the performance including the outcome of the next scheduled exam, treating the output of the network as a prediction. The results are visualized in Fig. 4 with the corresponding ROC curves. Overall, the results indicate that radiologists outperform the best-performing publicly available AI model in this screening population (*χ*^2^ > 800, *p* < 10^−10^ in all time points). Furthermore, for both the radiologist and the DMV model, the 3-year prediction task is more difficult than the diagnostic tasks in terms of sensitivity (*χ*^2^ = 180, *p* = 10^−40^)

**Fig. 5:**
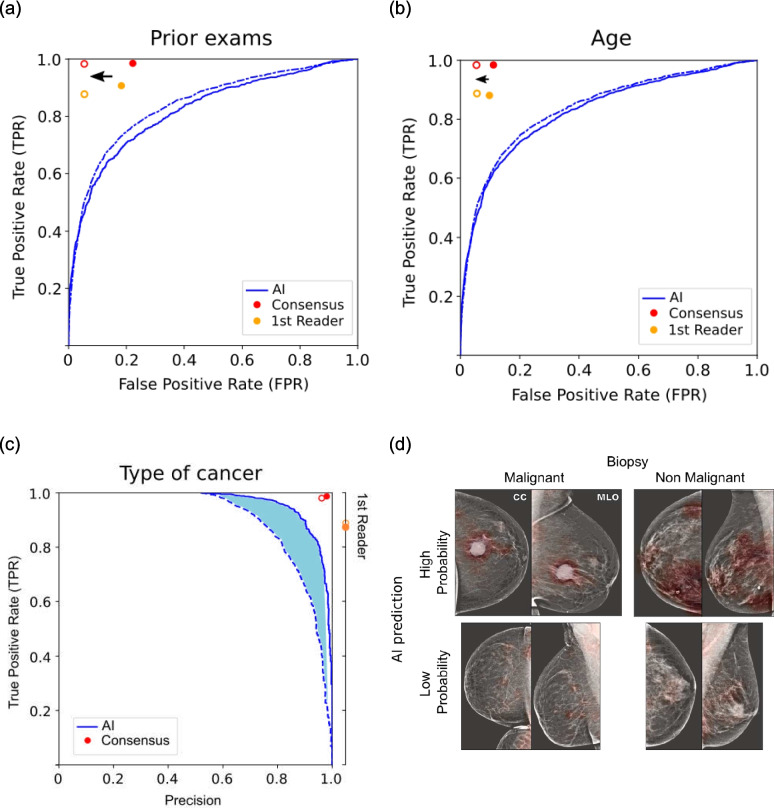
Comparison of breast cancer diagnosis performance between the AI model and clinical readers across different subgroups of the test set. **a** Exams were separated by type: first call to screening (solid lines and filled circle) or routine recall exam (dashed lines and unfilled circle). **b** Patients were separated by age: individuals older than 60 years (dashed lines and unfilled circles) and younger than 60 years (solid lines and filled circles). **c** True positive rate (TPR) vs precision of the AI model and clinical readers. Malignant cases were stratified by the type of breast cancer: in situ (solid lines) and invasive (dashed lines). **d **Heat maps (red shading) for examples of correct and incorrect classified images. The heat map indicates the probability of finding a malignant lesion at each location [15]

**Table 4:** Radiologist and network performance: performance is computed based on cancer confirmed within various follow-up intervals

Task		Diagnosis				Prediction
Time period		0–3 months screen detect	0–1 y interval	0–2 ys interval	0–3 ys interval	0–3 years + screen detect
1st reader	Sensitivity	0.88 ± 0.08	0.87 ± 0.08	0.86 ± 0.07	0.83 ± 0.08	0.64 ± 0.08
DMV	Sensitivity	0.54 ± 0.01	0.53 ± 0.01	0.52 ± 0.01	0.50 ± 0.01	0.42 ± 0.01
Both	Specificity	0.93 ± 0.02	0.93 ± 0.02	0.93 ± 0.02	0.93 ± 0.02	0.93 ± 0.02

### Factors that Influence the Performance

We analyze several factors that may limit the performance of the DMV and thus could lead to performance gains in future work.

#### Prior Exams

In routine clinical practice, radiologists often have access to the prior mammograms making it easier to determine if a lesion is new and abnormal. In contrast, the AI models tested here do not take prior mammograms into account. Indeed, we find that radiologists had better performance on the first call to screening as compared to subsequent routine calls (Fig. 5a). The relative number of false positives (FPR) for the first reader is reduced from 0.20 ± 0.07 in the first call to screening (filled yellow circle) to 0.05 ± 0.02 (*χ*^2^ = 1501, *p* < 10^−5^) in routine recalls for subsequent screening exams (unfilled yellow circle), maintaining the same TPR value (≃0.88).

#### Age

Radiologists may also take risk factors into account, such as age and family history, while none of the current models do. In Fig. 5b, the performance of the DMV model and the radiologists are shown for two subsets: patients older and younger than 60 years. Radiologist performance improved in the older population (FPR is reduced from 0.10 ± 0.04 to 0.05 ± 0.02; *χ*^2^ = 188, *p* < 10^−5^). We interpret this as a benefit of knowing the patient’s age because network performance, which would rely on changes in the actual images, does not seem to change.

#### Type of Cancer

An additional factor that may affect performance is the type of tumors. In situ breast cancer typically appears as small clusters of calcifications, while invasive breast cancer may appear as a mass with irregular borders or spiculated (star-like) projections [22]. We find that the network performs significantly better on in situ cancers as opposed to invasive cancers, whereas the performance of radiologists is essentially unchanged (Fig. 5c).

#### Location Information

When the training of the DMV network includes information on the location of the lesions, it learns to generate a heatmap (Fig. 5d). When this heatmap is not available, the network can still perform classification, but performance degrades, with AUC dropping from 0.84 ± 0.01 to 0.76 ± 0.01 [19].

### Analysis of False Negatives

**Fig. 6:**
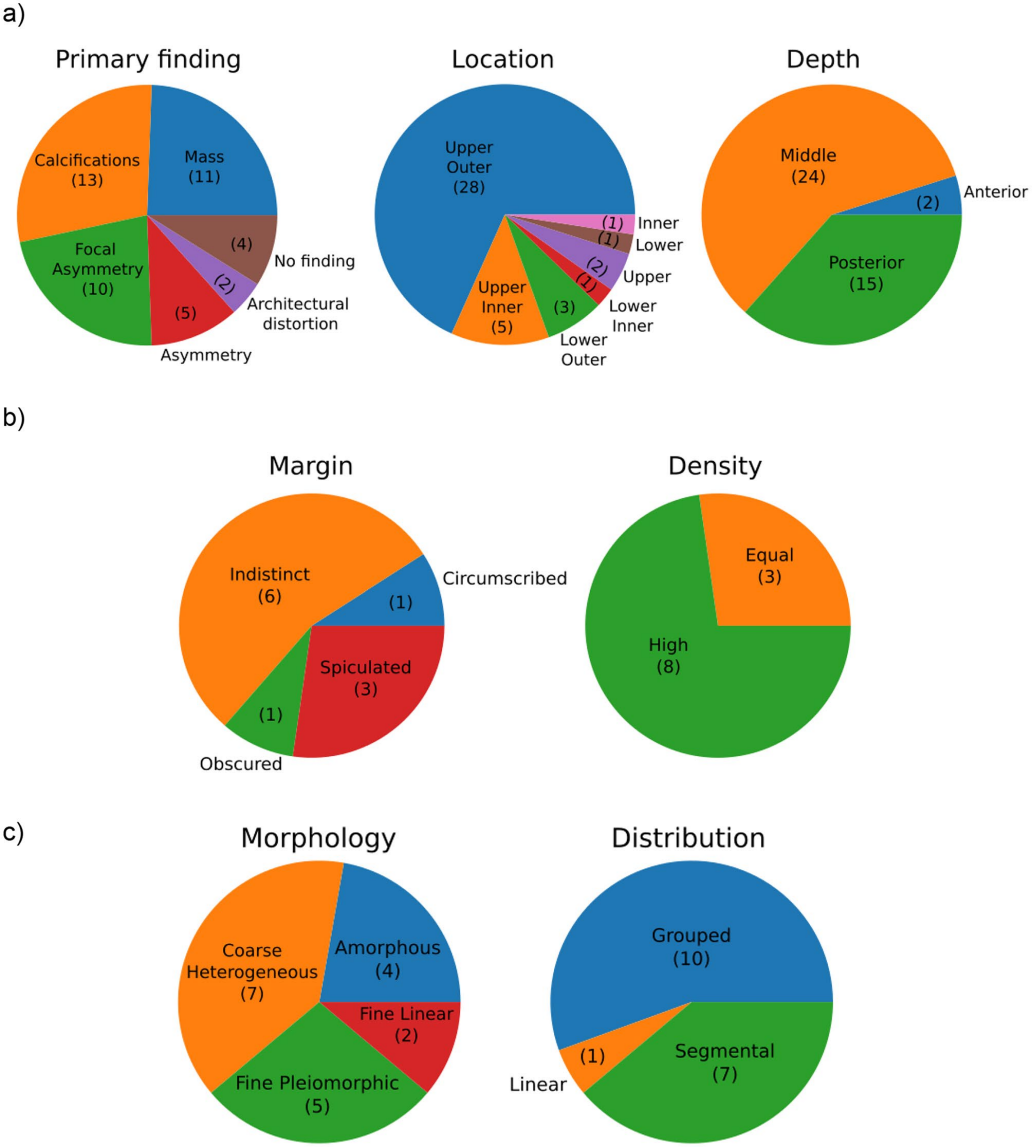
Characteristics of 45 cases missed by the DMV model. **a** Overall findings (*N* = 45). **b** Properties of masses (*N* = 11). **c** Properties of calcifications (*N* = 18)

A breast radiologist (CL) also inspected in more detail a subset of 45 malignant cases that the network failed to detect. These were false negatives with the lowest probability of malignancy and for which only a single view was available. On average, the size of the missed tumors measured 1.4 cm. The DMV model predominantly missed tumors with calcifications, followed by focal asymmetry and masses (Fig. 6a). This largely follows the prevalence of different kinds of cancers in this population. However, there is a lower proportion of masses in missed cancers (24%) as compared to the detected cancers (54%). In contrast, the asymmetries are more frequent in the missed cancers (11%) as compared to the detected cancers (0.6%). These differences in the distribution of detected and missed cancers are statistically significant (*χ*^2^ = 90.44, *p* = 1.07 × 10^−18^). This result suggests that the DMV model is relatively better at detecting masses, but it is not as proficient in identifying asymmetries or architectural distortions. The missed cases were frequently located in the upper outer breast region at middle depth (Fig. 6a, middle and right). This distribution of locations is consistent with values reported in the literature [23] (*χ*^2^ = 3.34, *p* = 0.18, e.g., 81% here vs. 68% in the literature for upper quadrants and 11% vs. 21% for lower quadrants). All of the missed masses had an irregular shape, with the most common margin being indistinct (Fig. 6b). Furthermore, the majority of these masses were labeled as high-density. Regarding the missed calcifications, which include cases where calcifications were an associated finding, the most common morphology observed was coarse heterogeneous (Fig. 6c). Additionally, the most frequent calcification distribution observed was grouped.

## Discussion

Recently, a number of reports documented impressive performance of deep-learning models in mammography diagnosis, at times matching the performance of trained radiologists [7–9, 13–16]. However, much of what has been published is difficult or impossible to reproduce, for a variety of reasons, e.g., the availability of the code, the availability of training data, or a lack of details about the training process. In this work, we made a concerted effort to independently evaluate all publicly available models on public datasets.

The classification performance was evaluated here using the AUC metric. The predictions obtained in our evaluation were consistent with the results previously published by Stadnick et al. [19]. This confirms that the published models are complete and that we successfully implemented their use, which is crucial when dealing with new and independent datasets.

On new data, models generally underperformed when using individual views. For the CBIS-DDSM dataset, the performance had previously been reported on a selected subset of the data [7]. When performance is evaluated on the complete set, all models dropped significantly in performance (from 0.85 to 0.7), suggesting that these data are more challenging. A possible cause for this may be that the distribution of image sizes and the intensity distribution are much wider for the CBIS-DDSM data as compared to the INbreast data (Fig. 2a and b). These results suggest that generalization will require large but also diverse datasets with high-quality data for training (i.e., harmonization of datasets). The alternative may be to use a narrowly defined acquisition protocol and scanner type, which is the approach taken by some commercial efforts (e.g., a model from Deep Health [9] operates only on mammograms collected with a Hologic scanner). At this point, we have limited ourselves to using the preprocessing defined in the original studies.

The models tested here differ in the way they integrate global and local image information as well as the ways of combining mammography views to predict benign and malignant lesions (Table1 and Fig. 3). A model that stands out with good generalization performance is the DMV model from NYU [15]. We attribute this to the combination of the two mammographic views from both breasts. In our view, this pre-trained model is a good starting point for further research in algorithm development, in particular as the code is available for further improvement and testing on new data. Therefore, further analysis focused on this model.

To conduct a comprehensive performance evaluation, we utilized data provided to us by the OPTIMAM project (OMI-DB dataset [17]). We evaluated diagnostic accuracy of the DVM model and the radiologists’ opinions based on pathology results. The model achieved an AUC of 0.84, but it fell short of matching the performance of individual radiologists [24]. At a specificity of 0.92, radiologists demonstrated a sensitivity of 0.97, whereas the network’s sensitivity was only 0.53 at the 1-year time point (Table 4 and Fig. 4). A limitation of our study is that this assessment of the radiologist’s opinion has a confirmation bias, i.e., cases deemed normal or benign were not independently evaluated. Only an unbiased reader study can really compare performance between radiologists and a network.

The results shown in Fig. 5a and b suggest that the radiologist’s analysis benefits from the availability of prior exams and risk factors, consistent with established literature [24]. This indicates that there are some opportunities for future AI models: (a) the implementation of priors and (b) the utilization of external domain-specific information (i.e., risk factors). There have been a few efforts in incorporating priors with mixed results [25, 26], and the effectiveness of incorporating risk factors is still in discussion [8, 27, 28]. McKinney et al. [8] argues that a model can operate at different levels of analysis depending on its focus: (a) low-level, individual lesions suggestive of cancer; (b) intermediate-level, the views of the same breast; and (c) high-level, the entire exam. The authors incorporate age information in the low-level analysis stage to reduce false negatives and false positives. However, Kooi et al. [28] found no significant improvements in their low-level model when adding age as a feature, suggesting that the relationship between age and cancer is challenging to learn at the individual lesion level due to its existence at the exam level. Lastly, Yala et al. [27] introduced a risk prediction model that leverages a high-level representation at the exam level to predict risk factors (e.g., age and breast density) in situations where these factors are unavailable, and this model exhibited enhanced risk discrimination compared to the Tyrer-Cuzick model.

A relevant aspect of the DMV model is the use of two class-specific heatmaps (malignant and benign) which are generated by the initial stage (called low-level classifier [29]). These heatmaps, which classify patches within the images, are then incorporated as additional input channels in the model’s second stage (called the main classifier). Through this approach, the model not only achieves breast classification but also generates interpretable heatmaps that indicate the locations of suspicious findings [15] (Fig. 5d). Note that training the low-level classifier requires the findings manually indicated on the images by radiologists [15]. Consequently, the conventional way to retrain the model on new data involves freezing the low-level classifier and updating only the main classifier. However, if the generated heat maps do not provide an accurate representation of lesion locations, it is advisable to retrain the low-level classifier whenever the necessary data is available (i.e., through radiologists’ segmentation).

As mentioned above, we have demonstrated that factors such as prior exams, patient age, type of cancer, and tumor location have an impact on the model’s performance. We have not analyzed other factors that may affect performance, such as differing vendor machines or harmonization, which may be a topic for future work.

In Figs. 5c and 6, we analyzed the failures of the DVM model as a function of different properties of the detected cancers. The results indicated that the AI model performs significantly better in identifying in situ lesions. More detailed analysis by a radiologist revealed that the networks tend to be relatively stronger at identifying cancers presenting as mass lesions and relatively weaker at identifying cancers presenting as asymmetries or architectural distortion. More analysis is needed to compare these trends to the relative strengths and weaknesses of radiologist cancer detection in an attempt to synergize the efforts of radiologists and deep learning.

## Conclusion

With the advancement of DL, the medical imaging community is interested in applying these techniques to improve the accuracy of cancer screening and the development of a new generation of CAD tools. This study is centered around the challenge of replication and generalization across diverse datasets, as well as the comparison of various available models. The analysis presented in this work shows that to ensure the safe and reliable use of available trained models, independent validation becomes an essential step. Moreover, future advancements in AI for mammography may benefit from a concerted effort to make larger datasets publicly available, encompassing multiple clinical sites. This will facilitate the development of more robust models that can generalize effectively across diverse populations and imaging settings, ultimately enhancing the performance and clinical utility of AI-assisted mammography screening.

### Supplementary Information

Below is the link to the electronic supplementary material.

Supplementary file1 (DOCX 20 KB)

## Data Availability

All test code will be made available at the time of publication on GitHub.

## Abbreviations

MLO: Mediolateral oblique
CC: Craniocaudal
DL: Deep learning
ML: Machine learning
CAD: Computer-aided detection
