# Supplementary material for: Robustness of Deep Networks for Mammography: Replication Across Public Datasets
Source: J Imaging Inform Med. 2024 Jan 10;37(2):536–46. doi: 10.1007/s10278-023-00943-5 (PMC11031505; doi:10.1007/s10278-023-00943-5)
Supplement: Supplementary file 1 — Supplementary file1 (DOCX 20 KB) [file 10278_2023_943_MOESM1_ESM.docx]

# Supplementary Information

**Datasets**

**CBIS-DDSM**

Digital Database for Screening Mammography (DDSM) is the result of the collaborative effort between the Massachusetts General Hospital, the University of South Florida, Sandia National Laboratories, Washington University School of Medicine, Wake Forest University School of Medicine, Sacred Heart Hospital and ISMD, Incorporated. DDSM is a database of 2620 exams and 10480 images of scanned film mammography studies. It contains normal, benign, and malignant cases with verified pathology information.

To solve different challenges presented by the original DDSM version for the evaluation of CAD systems research in mammography, Lee et al.[^11^](https://www.zotero.org/google-docs/?ZEouYU) developed an updated and standardized version of DDSM. The Curated Breast Imaging Subset of DDSM (CBIS-DDSM) collection includes a subset of the DDSM data selected and curated by a trained mammographer. An example of an update is that ROI segmentation and bounding boxes, and pathologic diagnosis for training data are also included. For details of the processing, it is recommended to read Lee et al[^11^](https://www.zotero.org/google-docs/?z5ljAA).

The dataset can be downloaded from TCIA website [^30^](https://www.zotero.org/google-docs/?nS5aDU). For this dataset, there is a standard separation of the training and test subsets. The test set consists of 349 exams (645 images), while the training set consists of 1248 exams (2458 images). As indicated in Fig. 2d, only 6 % of exams have the four views available.

**INbreast**

The INbreast database is a mammographic database whose images were collected at the Breast Centre of the Hospital de São João in Porto, Portugal, between April 2008 and July 2010. It has a total of 115 exams (410 images) of which 73 % of cases are from women with both breasts (4 images per case) [^10^](https://www.zotero.org/google-docs/?JWJ1bV).

The database includes examples of normal mammograms, mammograms with masses, mammograms with calcifications, architectural distortions, asymmetries, and images with multiple findings. The size of the images is 3328 x 4084 or 2560 x 3328 pixels.

INbreast dataset was released in 2012 and can be downloaded from Kaggle website [^31^](https://www.zotero.org/google-docs/?N4NTVg). There is no standard division of test and training subsets for INbreast. In this work, the dataset was not separated into subsets.

**CMMD**

The Chinese Mammography Database (CMMD) was collected between 2012 and 2016, in Sun Yat-sen University Cancer Center and Nanhai Hospital of Southern Medical University in Foshan. In 2021, it was published by The Cancer Imaging Archive (TCIA) and it can be downloaded from TCIA website [^32^](https://www.zotero.org/google-docs/?T1JXBE).

The database CMMD consists of 1775 patients/exams (5202 images): (1) 3728 mammographies with biopsy confirmed types of benign or malignant tumors (2) 1498 mammographies with additional information about molecular subtypes.

Dataset images are accompanied by biopsy-proven breast-level benign and malignant labels. Dataset authors also provided age and finding type (calcification, mass, or both) for all patients as well as immunohistochemical markers for 749 patients with invasive carcinoma.

**OMI-DB**

The OPTIMAM Mammography Image Database (OMI-DB)[^17^](https://www.zotero.org/google-docs/?w3hEST) is a large, publicly available dataset of digital mammography images that was created to support research and development of computer-aided detection (CAD) and computer-aided diagnosis (CADx) algorithms for breast cancer detection. The dataset contains over 100,000 images and accompanying clinical data, including information on the patient's age, breast density, and lesion pathology. The images were collected from screening and diagnostic mammography examinations conducted at three sites in the UK. The database also includes annotations of the mammographic abnormalities that were detected by radiologists. The OPTIMAM Mammography Image Database has been used in a wide range of studies evaluating the performance of different CAD and CADx algorithms, and it continues to be a valuable resource for researchers working in the field of breast imaging.

Of these data we receive the standard dataset shared with academics with a total of 6000 cases:

- 3500 malignant Cases, with expert annotations, and full clinical annotations
- 500 benign Cases, with expert annotations, and full clinical annotations
- 500 malignant Cases with full clinical annotations only
- 500 benign Cases with full clinical annotations only
- 1000 Normal Cases

The individual opinions are either ‘normal’, ‘abnormal’, ‘clinical’, ‘technical’ or ‘not applicable’. Abnormal indicates imaging evidence to recall the patient for further assessment, while ‘clinical’ indicates that the patient has symptoms or a radiographer notices a change during mammography prompting a recall. Technical recall means that the patient needs another screening due to technical issues, and “not applicable” is used when images can not be obtained, for instance, due to a prior mastectomy.

The decision to recall based on imaging relies on the consensus opinion as to whether the tissue appears ‘normal’, has ‘benign lesions’, ‘lesions of uncertain malignant potential’, ‘suspicious of malignancy’, or has ‘malignant lesions’. Anything other than normal or benign is recalled.

Finally, the exams also include a “closing date” indicating that immediate follow-up procedures have been completed, typically no more than 3 months.

**Details of AI models.**

The models analyzed were trained using a supervised learning approach. In the context of supervised learning, we consider three key elements: (1) a training dataset $D=\left\{ x_{n},y_{n} \right\}$, (2) a proposed model $F(x,\theta)$ with trainable parameters $\theta$ , and (3) a cost function $L(D,F)$ to minimize. This will be described next for each model.

**Convolutional Neural Networks (CNNs)**

One of the most relevant constructions of Machine Learning in recent years is the concept of Deep Convolutional Network. This idea is inspired by the anatomy and functionality of the visual system. In fractions of seconds, humans can identify objects within our field of vision. They can also name these objects, perceive their depth, perfectly distinguish their outlines, and separate them from their backgrounds[^33^](https://www.zotero.org/google-docs/?hiBRCB). Somehow the system captures pixel data and transforms that information into more meaningful features; e.g., geometry, abstractions, and semantic meaning. Convolutional neural networks (e.g. Resnet) are the result of a sequential grouping of different kernels. Generally, they are built of convolutional layers (convolutions/filters or blocks), reduction layers (pooling), and a fully connected classifier layer, which will give us the final result of the network (prediction).

**AI Models for Breast Cancer Diagnosis.**

In the models for the breast cancer diagnostic task, the input $X^{(n,s,v)}$ is an image for exam n, side s (left, right), and view v (MLO, CC), and the output $\hat{y}_{m}^{(n,s)}$ represents the probability of the presence of malignant for each breast s in the exam n. Each exam $E_{n}$ consists of one or more images, therefore we use the notation $E_{n}= \left\{ X^{(n,s,v)} \right\}$. Also, it has associated a set of binary labels $\left\{ y_{m}^{(n,s)} \right\}$ that indicate whether the breast $s$ of the exam $n$ presents a malignant lesion. Both the predictions and the labels are necessary for the calculation of the model's performance (i.e., AUC).

We studied different models (End2End, GLAM, GMIC, and DMV) with various architectures and training processes (see Table 5).

| **Model** | **CNN** | **Num**  **of parameters** | **Input** | **Output** | **Loss function** | **Training dataset** |
| --- | --- | --- | --- | --- | --- | --- |
| End2End  (MSSM) | Resnet-50  VGG16 | 23 M  134 M | One single  image | Probability of malignancy. | BCE between output and label | CBIS-DDSM  INbreast |
| GLAM  (NYU) | Resnet-22  (global network)  Resnet-34 (local network) | 21 M |  | Probability of:  1- malignancy.  2- benign lesion. | Sum of BCEs obtained in the different modules (global, local, and fusion). | NYU Breast Cancer Screening Dataset (186,816 exams with four views) |
| GMIC  (NYU) | Resnet-22 (global network)  Resnet-18 (local network) | 11 M |  |  |  |  |
| DMV  (NYU) | Resnet-22 | 21 M | One exam with 4 views: R-CC, L-CC, R-MLO, L-MLO | Probability of:  1- left breast malignancy.  2- right breast malignancy.  3- left breast  benign lesion.  4- right breast  benign lesion. | Sum of BCEs. |  |
| **Table 5: Information about the models analyzed in this work.** | | | | | |  |
